# The standardization of the report for urine cell counting—A converting factor for Sysmex UF‐1000i

**DOI:** 10.1002/jcla.22857

**Published:** 2019-03-27

**Authors:** Lina Wang, Huacheng Wang, Chanjing Zhao, Cha Chen

**Affiliations:** ^1^ Department of Laboratory Medicine Guangdong Provincial Hospital of Chinese medicine Guangzhou China; ^2^ Clinic Neuroscience Center, The Seventh Affiliated Hospital Sun Yat‐sen University Shenzhen China

**Keywords:** cell chamber, converting factor, flow cytometry, reference method, report unit, urine sediment

## Abstract

**Background:**

Multicenter laboratory may apply both automated flow cytometer and microscopy for urinalysis. Automated flow cytometer such as Sysmex UF‐1000i evaluates particles with native urine without centrifugation and reports as “counts per μL.” Microscopic examination recommended as the reference method for urine sediment analysis reports results as “counts per HPF (or μL).” Moreover, some results from flow cytometer are needed to be checked visually under microscopy. Therefore, it is worth to establish the consistency of the results from these two methods.

**Methods:**

Urine specimens from 412 patients were examined with Sysmex UF‐1000i and manual microscopy using FAST‐READ disposable counting chambers. White blood cell (WBC) and red blood cell (RBC) counting results from UF‐1000i after transferred with the converting factor (0.297) we estimated were compared with that from microscopic examination. Method comparison was performed using Passing‐Bablok analysis.

**Results:**

After transferred with the converting factor (0.297), cell counting results from UF‐1000i showed a good correlation with that derived by the reference method (*R* ^2^ was 0.868 for RBCs (*P* < 0.001), 0.882 for WBCs (*P* < 0.001)). Passing‐Bablok analysis showed no systematic difference (intercept estimate, −1 [95%CI, −7 to 3] and slightly proportional (slope estimate, 1.2 [95%CI, 1.0 to 1.7]) bias between concentrations of cells measured by manual microscopy and Sysmex UF‐1000i using the converting factor.

**Conclusion:**

The converting factor (0.297) helps to transfer “counts per μL (non‐centrifugal urine)” to “counts per μL (equal to centrifugal urine),” and to keep the urine particle analysis results of Sysmex UF‐1000i consistent with the results from the reference method.

## 1 INTRODUCTION

Routine urinalysis is frequently performed to help diagnosis and monitor urine tract and kidney diseases. Automated urinalysis flow cytometer such as Sysmex UF series has been adopted in clinical laboratory for almost two decades.^1^ When reporting the cell counts, the UF‐1000i system provides two different kinds of units, which are “cells per μL (non‐centrifugal urine)” and “cells per HPF (by calculated automatically).” The value of UF series is obviously higher than those obtained with microscopic examination,^2^ which brings confusion to clinicians when they are not used to the units of UF series.^3^

The microscopic urine sediment analysis is recommended as the reference method for the urine cell analysis.^4^, ^5^ The sample showing abnormalities or no coincident between the flow cytometer and the dipstick was re‐examined under the microscopy using cell chambers (KOVA or FAST‐READ etc). The guideline GP‐163A^4^ approved by Clinical and Laboratory Standards Institute (CLSI) mentioned that the urinalysis results should be reported in the same reporting format and using the same reference intervals, and emphasized that the cell counts from manual microscopy are not suitable for comparison between laboratories. However, Chinese expert consensus^5^ required that results from different methods‐based analyzers should be reported with their own reference interval and proposed that the urine sediment results should be reported as “counts per HPF (centrifugal urine).” In summary, the agreement has not been reached of these guidelines on the unit for reporting urine sediment results. The various reporting forms for the same test may bring confusion to clinicians who evaluate the treatment effect for patients with kidney disease on the basis of urine sediment results.

We adopted a converting factor to make the results from UF‐1000i comparable to the results from recommended urine sediment examined procedure. After that, we testified this converting factor by an experiment.

## 2 MATERIALS AND METHODS

### 2.1 Specimens

A total of 412 fresh urine specimens were obtained from inpatients of Guangdong Provincial Hospital of Chinese Medicine during a period of 3 weeks. The samples were collected in polypropylene containers. The analysis was performed on the UF‐1000i (Sysmex Corporation, Kobe, Japan) and then by FAST‐READ disposable counting chambers (Immune Systems Ltd, Paignton, UK). This whole process was finished within 1 hour of receipt.

### 2.2 Centrifugation efficiency estimation

The percentage of particles remained in supernatant or lost by discard could only be estimated. Fifty‐five urine specimens were randomly selected for centrifugation efficiency estimation. After analyzed on the UF‐1000i for the first time, samples were centrifuged for 5 minutes at 400 *g*. The supernatant was absorbed into a new tube (leaving about 200 μL residue) and mixed by reversing the tubes for eight times and then measured by UF‐1000i again. The centrifugation efficiency was calculated by the equation below:

(1) Efficiencyfactor(EF)=1-(concentrationinsupernatant/concentration innativeurine)

### 2.3 Adjustment of the UF‐1000i converting factor

According to the recommended procedure of urine sediment analysis,^4^ the sediment samples were examined without staining, and the RBC and WBC enumeration results were reported as average particle counts per high‐power field (HPF, ie, 1 field at 400× magnification, 10× 0bjective).

The volume of the urine in per high power field (HPF) depends on the aliquot pipetted the microscope slide, the diameter of the field of view and the side length of the cover glass. The cells were concentrated 50 times when 200 μL residential sediment from 10 mL original urine was left for cell quantitative account after centrifuge. According to the estimation by Hannemann‐Pohl et al,^6^ 20 μL sediment of the suspended 200uL pellet was added to a slide and covered with a 18 × 18 mm coverslip; thus, the thickness (height) of the suspension turns to be 0.0617 mm. Considering that the diameter of the view of the HPF was 0.5 mm, the cylinder‐shaped volume was about 0.0121 μL (*V* = (*d*/2)^2^ ∗π∗h). At the beginning, almost 10ml native urine was centrifuged generating 200μL sediment, which means that the native urine was 50 times concentrated. Thus, we can estimate that the result of particles per HPF from the reference method (centrifuged urine) equals the particles in 0.606 μL of non‐centrifugal urine.

The reference method assumed that all the particles were in the 200 μL sediment after centrifuge without any dismiss, which means that EF(Ref) was 100%. However, the manufactory of UF‐1000i considered that some of the particles are destroyed during centrifugation or adhere to the wall of the tubes. Thus, according to their manual, cells per HPF reported by UF‐1000i only represents the cells in 0.18uL original urine. They held the view that other cells in the rest of 0.426 uL original urine may have been lost during centrifuge. Therefore, the UF‐1000i assumed centrifuge efficiency related to the reference method should be 29.7% (Equations 2 and 3).

(2) $$\text{EF}\;\left(\text{UF}-\text{1}000\text{i},\text{assumed}\right)/\;\text{EF}\left(\text{Ref}\right)=0.\text{18}/0.\text{6}0\text{6}$$

(3) $$\text{EF}\;\left(\text{UF}-\text{1}000\text{i},\;\text{assumed}\right)\;=0.\text{18}/0.\text{6}0\text{6}\;*\text{EF}\left(\text{Ref}\right)=0.\text{297}*\text{1}00\%=\text{29}.\text{7}\%$$

### 2.4 Urine particle analysis with Sysmex UF‐1000i analyzer and microscopy

The urine samples was centrifuged at 400 *g* for 5 minutes, then the supernatant was discarded and the 200 µL of residual sediment was suspended. 10 μL of the suspended 200 μL pellet was placed into the FAST‐READ chambers, and the particle counting results were expressed as cells/μL.

The results of Sysmex UF‐1000i WBC and RBC counts of each sample were recorded after converting with Equation 4 and then were compared with that from microscopic examination.

(4) $$\text{Particles}/\mu \text{L}\;\left(\text{equal to centrifugal urine}\right)\;=\;\text{Particles}/\mu \text{L}\;\left(\text{non}-\text{centrifugal urine}\right)\;*\;\text{EF}\;\left(\text{UF}-\text{1}000\text{i},\;\text{assumed}\right)$$

### 2.5 Statistical analysis

The SPSS statistical 20.0 software (IBM, Armonk, NY, USA) was used to analyze the EF results and to do regression analysis between the results from UF‐1000i and chamber methods. EF data were presented as X50% (X25%, X75%). Passing‐Bablok analysis was also performed to make comparison between UF‐1000i and chamber methods (after conversion).

## 3 RESULTS

### 3.1 Efficiency of centrifugation

**Figure 1:**
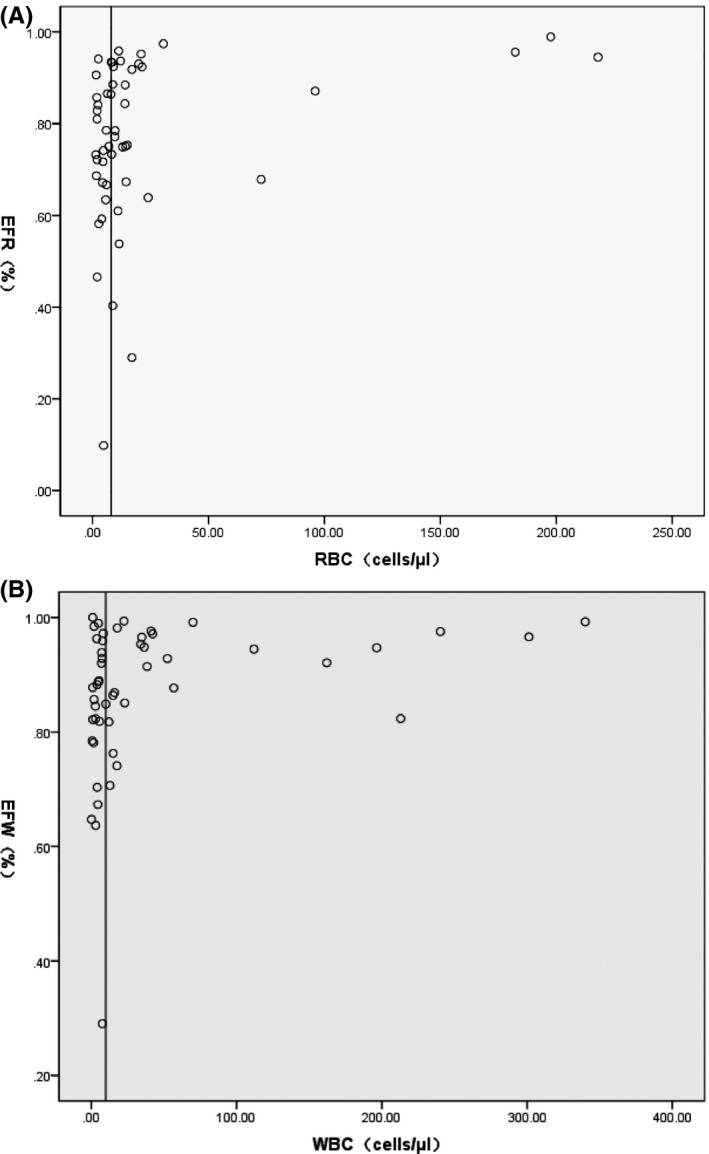
Urine centrifuge efficiency factor under different cell concentrations. (A) EFW (efficiency factor of WBC) and (B) EFR (efficiency factor of RBC) were calculated by Equation 1. The vertical bar represents the cell concentration of the reference limit (RBC counts were 6 cells/μL; WBC counts were 10 cells/μL)

The efficiency of centrifugation is presented in Figure 1. Around the concentration of the reference limit, the efficiency factor of RBC (EFR) was 77.8% (10%, 99%), and the efficiency factor of WBC (EFW) was 88.9% (29%, 100%). The EF was relatively high at the high concentration of cells. The centrifuge efficiency should be carefully considered.

### 3.2 Comparison results between FAST‐READ chambers and UF‐1000i after conversion

Regression analysis was performed for results (WBC and RBC) from chamber counting and UF‐1000i. When compared, all the results were converted to counts per µl, and the cell counts from UF‐1000i were transferred using Equation 4.

**Figure 2:**
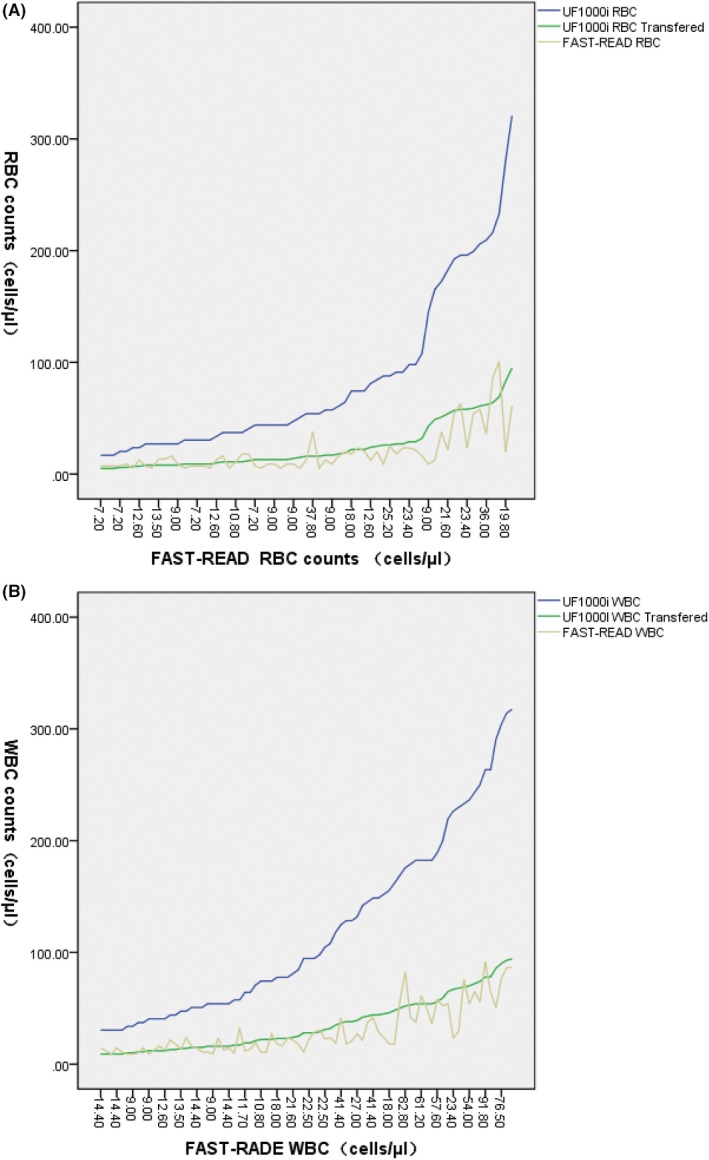
The consistency of UF‐1000i cell count (after conversion) with FAST‐READ chamber. (A) Red blood cell and (B) white blood cell (WBC) were measured by UF‐1000i compared to FAST‐READ chamber under microscopy after centrifugation. The results from UF‐1000i were converted using Equation 2. The data were plotted sequential as the UF‐1000i results increased

When the cells counts in urine below the upper limit of the reference interval or above 100, the actual number of those cells is insignificant according to clinical practice, even if these results do have clinical diagnosis meaning. RBC counts of 65 samples between 6 and 100 cells/μL (both on FAST‐READ and on UF‐1000i after conversion), and WBC counts of 78 samples between 9 and 100 cells/μL (both on FAST‐READ and on UF‐1000i after conversion) from the tested sample are presented in Figure 2. We observed a satisfactory correlation between UF‐1000i and FAST‐READ chamber counting (after conversion) using regression statistics. To be more specific, for RBC, *R* ^2^ was 0.868 with *P* < 0.001; for WBC, *R* ^2^ was 0.882 with *P* < 0.001.

In addition, comparison between UF‐1000i and chamber counting (after conversion) was made using Passing‐Bablok analysis. The estimates of linear regression functions (UF‐1000i results converted by Equation 4 vs concentration in urine from manual microscopy of sediment) for RBC and WBC are shown in Table 1. The 95% confidence range for the intercept of the regression lines contains 0, which means there is no systematic difference between UF‐1000i (using the converting factor) and chamber counting. The slopes of the estimated linear functions, at around 1.2, are very close to 1.0, indicating a good correlation between two methods.

**Table 1:** UF‐1000i (after conversion) compared to microscopy by Passing and Bablok analysis

	Slope *A*	95% confidence range	Intercept *B* (/μL)	95% confidence range	Number of samples
RBC	1.2698	1.0370 to 1.6667	−0.1429	−7 to 2.7333	65
WBC	1.2082	1.0819 to 1.4021	−1.1791	−5.1190 to 2	78

*Y* = results of UF‐1000i, using Equation 4 (particles/μL); *X* = results of manual microscopy of sediment (particles/μL); according to the following equation: *Y* = *AX* + *B*.

## 4 DISCUSSION

Sysmex UF‐1000i (TOA Medical Electronics, Kobe, Japan) flow cytometer makes urine particle counting more precise and effective. Several guidelines recommended that negative results of WBC or RBC from urine strips can be reported without checking by manual microscopy, and optimizing workflow also helps to decrease the microscopic examination of the samples with positive results of urine strips.^1^ A few laboratories have amended their own criterions^7^ and make them suitable for joint detection of urine flow cytometer and urine dipstick to reduce manual urine microscopy analysis.

The reference procedure of the International Society of Laboratory Hematology (ISLH) recommended using native urine for the quantitative urine particle analysis.^8^ However, if the amount of the clinically significant elements was too small in the urine, they may be missed.

Although we can use the same unit (cells/μL) to evaluate the cells in both centrifugal and non‐centrifugal urine, the results of the same sample on these two conditions and clinical significance are obviously different. Centrifugation and discarding steps are the main error source of manual microscopy examination. Although the efficiency of centrifugation can be increased significantly by tripling the centrifugation, there is an increase in the particle destruction rate at the same time. Therefore, the particles in the supernatant do not mean that the number of particles in the sediment is decreased. Taken this into account, it is not surprised that comparison experiments showed that the UF series urine flow cytometer (Sysmex) may detect more RBCs and WBCs than microscopic examination did.^9^ This finding brought confusion to clinicians in hospital which has multicenter laboratories applying both urinary sediment microscopic examination and flow cytometry analyzer. Simultaneously utilizing the UF‐1000i analyzer, FAST‐READ chamber in our laboratory has already confused clinicians in our hospital.

The converting factor (0.297) we introduced here makes the original counts per μL (non‐centrifugal urine) transferred to “counts per μL (equal to centrifugal urine),” and enables the urine particle analysis results of Sysmex UF‐1000i correlated with that from chamber counting. The intercept of the regression lines found with the Passing‐Bablok analysis is a negative value, which means that the UF‐1000i method (after conversion) may have bias when the concentration of WBC or RBC is <1 cells/µL. The slopes of those regression lines, which are really close to 1, showed after conversion UF‐1000i counting is comparable to the reference method.

According to Hannemann‐Pohl et al,^6^ the WBC count in native urine measured by UF series analyzer is 3.15 times of that derived from urine sediment analysis, which is very close to our converting factor (1/0.297 = 3.37).

Since the concentration of urine particles needs to be continuously monitored, it is better to keep the same reporting form for urine particle result. Thus, using the same reference intervals in a certain laboratory is more convenient.
